# Extraction of Protein Interaction Data: A Comparative Analysis of Methods in Use

**DOI:** 10.1155/2007/53096

**Published:** 2007-12-09

**Authors:** Hena Jose, Thangavel Vadivukarasi, Jyothi Devakumar

**Affiliations:** 1 Jubilant Biosys Ltd., #96, Industrial Suburb, 2nd Stage, Yeshwanthpur, Bangalore 560 022, India

## Abstract

Several natural language processing tools, both commercial and freely available, are used to extract protein interactions from publications. Methods used by these tools include pattern matching to dynamic programming with individual recall and precision rates. A methodical survey of these tools, keeping in mind the minimum interaction information a researcher would need, in comparison to manual analysis has not been carried out. We compared data generated using some of the selected NLP tools with manually curated protein interaction data (PathArt and IMaps) to comparatively determine the recall and precision rate. The rates were found to be lower than the published scores when a normalized definition for interaction is considered. Each data point captured wrongly or not picked up by the tool was analyzed. Our evaluation brings forth critical failures of NLP tools and provides pointers for the development of an ideal NLP tool.

## 

[1,2,3,4,5,6,7,8,9,10,11,12,13,14,15,16,17,18]
